# Exploratory Associations of Personality Traits, Cognitive Emotion Regulation, and Quality of Life with DSM-Related Symptom Burden in Gambling Disorder

**DOI:** 10.3390/clinpract16070122

**Published:** 2026-06-29

**Authors:** Ioana Ioniță, Mădălina Iuliana Mușat, Bogdan Cătălin, Dan Adrian Lutescu, Constantin Alexandru Ciobanu, Adela Magdalena Ciobanu

**Affiliations:** 1 “Carol Davila” Doctoral School, Faculty of Medicine, University of Medicine and Pharmacy, 050037 Bucharest, Romania; ioana_ionita@ymail.com; 2 Experimental Research Center for Normal and Pathological Aging, University of Medicine and Pharmacy of Craiova, 2 Petru Rareș Street, 200349 Craiova, Romania; 3 Department of Scientific Research Methodology, University of Medicine and Pharmacy of Craiova, 2 Petru Rareș Street, 200349 Craiova, Romania; 4 Department of Physiology, University of Medicine and Pharmacy of Craiova, 2 Petru Rareș Street, 200349 Craiova, Romania; 5 Department of Medical Informatics and Biostatistics, Faculty of Dentistry, “Carol Davila” University of Medicine and Pharmacy, 050037 Bucharest, Romania; dan-adrian.lutescu@drd.umfcd.ro; 6 Faculty of Medicine, “Carol Davila” University of Medicine and Pharmacy, 020022 Bucharest, Romania; alexandru.ciobanu2020@stud.umfcd.ro; 7 Department of Psychiatry, “Prof. Dr. Alexandru Obregia” Clinical Hospital of Psychiatry, 041914 Bucharest, Romania; adela.ciobanu@umfcd.ro; 8 Neuroscience Department, Discipline of Psychiatry, Faculty of Medicine, “Carol Davila” University of Medicine and Pharmacy, 020021 Bucharest, Romania

**Keywords:** gambling disorder, personality traits, emotion regulation, CERQ, quality of life, PCF, DSM-5, addiction, psychopathology, coping strategies

## Abstract

**Background and Objectives:** Gambling disorder (GD) is a behavioral addiction associated with distress, comorbidity, and functional impairment. This exploratory cross-sectional study examined associations between DSM-5-TR symptom burden, personality dimensions, cognitive emotion regulation, quality of life, and sociodemographic variables in a Romanian clinical sample. **Materials and Methods:** The sample included 122 adults with psychiatrist-confirmed pathological gambling/GD recruited from “Prof. Dr. Alexandru Obregia” Clinical Hospital of Psychiatry, Bucharest. Personality was assessed with the Personality Clinical Form (PCF; 109 valid profiles), cognitive emotion regulation with the Cognitive Emotion Regulation Questionnaire, and quality of life with the Quality of Life Inventory. Symptom burden was measured using a nine-item binary DSM-5 symptom burden index. **Results:** The symptom burden index showed a pronounced ceiling effect: median = 9.00 (IQR = 9.00–9.00; range = 4–9), with 91.0% classified as severe and 77.9% meeting all nine criteria. In PCF analyses, symptom burden was positively associated, after Benjamini–Hochberg correction, with broad personality pathology, including maladaptive personality dimensions, personality-functioning indicators, and personality-disorder feature scales; the strongest association involved borderline features. Catastrophizing and Blaming Others were positively associated with severity, whereas Positive Reappraisal, Putting into Perspective, and Positive Refocusing were negatively associated. Quality of life was very low overall and associated with personality and coping variables, but not directly with symptom burden. Criterion-count rank distributions differed by marital status and perceived social support; occupational status showed an omnibus distributional difference, but no pairwise contrast survived correction. **Conclusions:** GD was characterized by severe symptom burden and restricted score variability. Findings support multidimensional assessment of personality functioning, emotion regulation, quality of life, and social–contextual vulnerability.

## 1. Introduction

Gambling disorder (GD) is a complex psychiatric condition characterized by persistent and recurrent maladaptive patterns of gambling behavior, leading to significant psychological distress and functional impairment [1]. Recent public-health sources estimate that approximately 1.2% of the global adult population has gambling disorder, while broader categories of at-risk or problematic gambling yield higher estimates [2,3]. In Romania, epidemiological data on gambling behavior remain limited. A nationally representative market-research survey of 988 adult individuals showed 15% of respondents had gambled at least once in the previous 12 months, and 0.6% of the population—approximately 98,000 individuals—were classified as problem/pathological gamblers [4]. Studies on adolescents suggest a higher burden in younger populations: 7.1% at-risk gambling and 4.0% problem/pathological gambling using the South Oaks Gambling Screen—Revised for Adolescents (SOGS-RA), and 10.1% problem gambling and 2.6% pathological gambling using the 20 Questions of Gamblers Anonymous - Revised for Adolescents (20 GA-RA) [5].

In psychiatric nosology, GD is the preferred diagnostic term, though partially overlapping labels remain in use and reflect different historical, clinical, and methodological perspectives. Pathological gambling was mainly used in earlier DSM-IV/ICD-10 terminology, whereas gambling addiction emphasizes the current addiction framework [6,7]. Compulsive gambling is primarily a historical or lay expression describing repetitive gambling with subjective loss of control, while problem gambling is more commonly used as a public-health and screening construct [8,9,10]. Consistent with continuum models, gambling involvement can be operationalized in population studies using the Problem Gambling Severity Index (PGSI), ranging from non-problem or recreational gambling (PGSI = 0) to low-risk gambling (PGSI = 1–2), moderate-risk gambling (PGSI = 3–7), and problem gambling (PGSI ≥ 8) [10,11,12,13]. These categories indicate risk of gambling-related harm, not diagnoses. 

In 2013, with the publication of the DSM-5, pathological gambling was renamed gambling disorder and reclassified from the DSM-IV category of impulse-control disorders to the DSM-5 chapter on substance-related and addictive disorders. This is reflective of clinical, phenomenological and neurobiological similarities with substance use disorders and share comorbidity-related and treatment-related similarities [14]. GD is frequently associated with psychiatric comorbidity, particularly substance use disorders, alcohol use disorders, anxiety and mood disorders, suicidality, attention-deficit/hyperactivity disorder, impulsivity and broader emotional dysregulation [14,15]. Studies on pathological gamblers highlight relevant risk-factors such as young age, male sex, early exposure to gambling, living alone, limited social support, financial strain, availability of online-gambling, impulsivity, maladaptive coping strategies, and gambling-related cognitive distortions [15]. These comorbidities may reflect broader psychopathological distress and clinical burden.

The clinical expression of GD is highly heterogeneous and may reflect the interaction between personality structure, cognitive emotion regulation processes, and contextual or environmental influences [16]. Current evidence suggests that maladaptive personality traits, particularly those related to negative emotionality, disinhibition, and impulsivity are frequently observed in individuals with GD and may be clinically relevant for multidimensional assessment and case formulation [17,18,19,20]. Cognitive emotion regulation strategies, as measured by instruments such as the Cognitive Emotion Regulation Questionnaire (CERQ), have also been associated with gambling-related problems [21,22,23]. Moreover, previous findings suggest a paradoxical pattern in which strategies usually considered adaptive, such as positive reappraisal, may sometimes be used by gamblers to reinforce self-serving interpretations of gambling outcomes [21]. Quality of life represents another important dimension, as gambling-related harms may affect interpersonal relationships, occupational functioning, financial stability, and psychological well-being [24,25]. 

This study aims to provide an exploratory multidimensional analysis of GD by examining the associations between clinical, psychological, and sociodemographic variables in a Romanian psychiatric sample. Specifically, the objectives of the study are:

O1—To analyze the correlations between personality dimensions assessed using the Personality Clinical Form (PCF) and DSM-related gambling symptom burden evaluated through a semi-structured clinical interview;

O2—To investigate the associations between cognitive emotion regulation strategies assessed using the CERQ and DSM-related gambling symptom burden;

O3—To examine the relationships between quality of life, assessed using the QOLI, and both personality dimensions and cognitive emotion regulation strategies;

O4—To explore the direct association between quality of life and DSM-related gambling symptom burden; and

O5—To assess whether DSM-related gambling symptom burden differs according to selected sociodemographic and contextual variables.

Based on previous literature findings, the following exploratory hypotheses were formulated:

**H1.**  *Higher DSM-related gambling symptom burden would be positively associated with PCF psychopathological tendencies, maladaptive personality domains, maladaptive traits, personality-functional impairment, and personality disorder feature scores;*

**H2.**  *Higher DSM-related gambling symptom burden would be positively associated with less adaptive CERQ strategies and negatively associated with more adaptive cognitive emotion regulation strategies;*

**H3.**  *Lower quality of life would be associated with higher maladaptive personality scores and less adaptive cognitive emotion regulation patterns, while the direct relationship between quality of life and gambling symptom burden was examined exploratorily;*

**H4.**  *DSM-related gambling symptom burden would differ according to sociodemographic and contextual variables;*

By integrating these dimensions within a single analytical framework, the study seeks to contribute to a more nuanced understanding of the psychological correlates of gambling-disorder symptom burden in a treatment-seeking clinical sample.

## 2. Materials and Methods

### 2.1. Study Setting, Recruitment and Ethics

This exploratory observational cross-sectional study was conducted at the “Prof. Dr. Alexandru Obregia” Clinical Hospital of Psychiatry, Bucharest, Romania. Potentially eligible participants were identified, after ethics approval, through the hospital database among adult patients admitted to inpatient units or day-hospital services between 1 October 2023 and 31 January 2026 with a clinical diagnosis of ICD-10 F63.0 Pathological Gambling, yielding a total of 251 cases. The Ethics Committee approval covered both retrospective screening of hospital records and prospective recruitment from the hospital database. Patients admitted before the ethics approval date were identified retrospectively from clinical records and contacted for participation only after approval, whereas patients admitted after approval were approached prospectively during inpatient or day-hospital care. Participants contact and enrolment began on 8 December 2025. No psychometric research assessment was performed before ethics approval or before documented informed consent. The final study sample comprised 122 adult participants who met the following inclusion criteria: (1) age 18 years or older; (2) provision of informed consent before any study-related procedure; (3) sufficient capacity to understand the study requirements, as judged by the investigator; and (4) a psychiatrist-confirmed clinical diagnosis of pathological gambling/gambling disorder according to the diagnostic criteria in force at the time of clinical assessment. The exclusion criteria were: (1) absence of a formal diagnosis of pathological gambling/gambling disorder; and (2) a current diagnosis of intellectual disability/intellectual developmental disorder. 

A participant was considered a study completer if the psychological assessment battery required for the planned analyses was completed. Complete-case analysis was used for scale-level analyses; therefore, participants with missing responses on a given instrument were excluded only from analyses involving that instrument. The difference in sample size reflects instrument-specific validity screening rather than participant exclusion from the study. Thirteen respondents produced Personality Clinical Form (PCF) profiles that did not meet the instrument’s validity requirements and were, therefore, excluded from PCF-based analyses only. Because profile validity pertains exclusively to the interpretability of PCF scores, these cases remained eligible for all analyses involving DSM-5 criteria, CERQ, QOLI, and sociodemographic variables, when complete data were available. This approach follows standard psychometric methodology and maximizes data retention while maintaining the validity of personality-related analyses. Accordingly, PCF-based analyses were conducted in the PCF-valid subsample of 109 participants, whereas non-PCF analyses were conducted in the full sample of 122 participants whenever complete data were available. Identifiable information was coded and stored separately from the analytical dataset. This study was conducted in accordance with the Declaration of Helsinki and was approved by the local Ethics Committee of the “Prof. Dr. Alexandru Obregia” Clinical Hospital of Psychiatry, Bucharest, Romania (approval no. 35410/4 December 2025). All participants provided informed consent before completing any study-related questionnaire. A detailed flowchart of the study design is provided in Supplementary Materials S1—Figure S1.

### 2.2. Diagnosis and Clinical Interview

Initial diagnostic status was established clinically by a qualified psychiatrist according to the diagnostic criteria in force at the time of clinical assessment, including ICD-10 F63.0 Pathological Gambling and/or DSM Gambling Disorder criteria. At study inclusion, all potentially eligible participants underwent diagnostic screening with the Structured Clinical Interview for DSM-5 Disorders–Clinician Version (SCID-5-CV), administered by the principal investigator. The SCID-5-CV initial evaluation and current-disorders screening section was used to support eligibility assessment and to screen for possible current psychiatric symptoms. However, positive screening responses were not followed by full SCID-5-CV diagnostic modules for all comorbid disorders; therefore, the study did not generate systematic structured diagnoses of psychiatric comorbidities. Within this screening section, item I16 was used as a gateway item for gambling involvement during the previous 12 months. An affirmative response to item I16 was not considered diagnostic by itself but prompted a focused DSM-5 criteria-based assessment of gambling disorder covering the nine diagnostic criteria [6]. 

Symptom severity was assessed using a structured clinical interview based on the nine diagnostic criteria for Gambling Disorder as specified in DSM-5-TR [26]. Each criterion was evaluated through standardized interview questions and rated as present (1) or absent (0), consistent with the binary diagnostic logic of the DSM-5 classification system. A criterion was considered present if the participant endorsed the corresponding behavior at any frequency – from Rarely to Very Often. The total symptom burden index was computed as the sum of criteria met (range: 0–9), with higher scores reflecting greater symptom burden. Severity categories followed DSM-5 specifications: mild (4–5 criteria), moderate (6–7 criteria), and severe (8–9 criteria). The internal consistency of the nine-item binary symptom burden index was examined using Cronbach’s alpha on the full sample (*N* = 122). Corrected item-total correlations were computed using Spearman’s rank-order coefficient to account for the binary response format. Criterion prevalence (proportion of participants meeting each criterion) is also reported. The results are presented in Supplementary Materials S2—Table S1.

The overall internal consistency of the binary score was modest-to-acceptable (Cronbach’s α = 0.650). C1 could not be evaluated using corrected item-total correlation because it was endorsed by all participants and therefore had zero variance. Among the remaining eight estimable items, seven showed statistically significant corrected item-total correlations. C6 (Chasing losses) showed a non-significant item-total correlation (r_it = 0.151, *p* = 0.096) but was retained because it constitutes a DSM-5-TR diagnostic criterion. The modest alpha value should be interpreted in the context of the pronounced ceiling effect: 77.9% of participants met all nine criteria, resulting in very limited score variance.

The clinical research assessment also included demographic data, namely age, gender, marital status, educational status, occupational status, monthly income, and area of residence, as well as family history of psychiatric or addictive disorders, perceived social support, age at gambling onset, type of gambling activity, gambling frequency, average session duration, and psychometric assessment. Identifiable information, including names and contact details, was coded to preserve confidentiality and stored separately from the analytical dataset.

All questionnaires were administered by the principal investigator, who was licensed to administer the psychometric instruments used in the study. Hospitalized participants completed the assessment battery on site in a paper-and-pencil format. Participants who completed the assessment outside the hospital setting received a Google Forms link containing the same items, wording, response options, and administration instructions as the paper version. Scoring and interpretation were performed separately, in accordance with the manuals and licensed procedures for each instrument.

### 2.3. Psychometric Instruments

*The Personality Clinical Form* (PCF; license no. 0845) was used to characterize participants’ clinical personality profile [27,28,29]. The PCF is a Romanian self-report personality inventory developed within the DSM-5 framework to assess dysfunctional personality traits, maladaptive personality domains, personality-functioning impairment, major psychopathological tendencies, and categorical personality-disorder features. The instrument includes 200 dichotomous True/False items and generates more than 50 scores, including validity indicators designed to detect inconsistent responding, infrequent responding, self-disclosure style, experienced distress, socially desirable responding, and defensive responding. In the present study, PCF outputs were generated using the licensed scoring procedure, and calibrated T-scores were used for all analyses and clinical interpretation. Protocol validity was evaluated according to the instrument’s validity indicators; only valid PCF profiles were included in PCF-based analyses. The PCF was used for dimensional clinical characterization of personality-related variables and not as a stand-alone diagnostic instrument for personality disorders. A detailed list of PCF scales and abbreviations is provided in Supplementary Materials S3.

According to the PCF manual [27], the instrument was standardized on a large Romanian sample (*N* = 1400), with additional validation analyses conducted in non-clinical, clinical outpatient, and test–retest subsamples. The reported internal consistency was adequate: Cronbach’s alpha ranged from 0.62 to 0.82 for the 26 primary clinical trait scales, with a median of 0.71, and from 0.70 to 0.93 for the composite/derived scales, with a median of 0.81. Test–retest stability over a two-month interval was also acceptable, ranging from 0.55 to 0.84 for primary trait scales and from 0.68 to 0.90 for composite scales. Construct validity and comparability with internationally used personality instruments were examined through convergent analyses with established measures, including the PID-5, SCID-5-PD-SPQ, OMNI-IV, MCMI-III, MMPI-2, DAPP-BQ, LPFS-BF, and the Outcome Questionnaire. The strongest comparability evidence was reported for the PID-5: 19 of the 25 homonymous PCF–PID-5 trait correlations exceeded 0.60, five exceeded 0.50, and in 23 of 25 cases the highest association was observed with the corresponding homonymous PID-5 scale. The categorical personality-disorder scales also showed meaningful convergence with established international instruments, with median homonymous correlations of 0.69 with OMNI-IV, 0.69 with MCMI-III, and 0.71 with SCID-5-PD-SPQ. Therefore, although the PCF is a Romanian instrument rather than a direct translation of an international scale, its validation data support adequate reliability, construct validity, and meaningful comparability with internationally used dimensional and categorical personality-assessment instruments.

Because PCF scoring was performed using licensed software-generated calibrated T-scores, current-sample internal consistency could not be independently recalculated from the exported score outputs; therefore, psychometric support was based on the instrument documentation and published validation evidence [28,29].

The Cognitive Emotion Regulation Questionnaire (CERQ; license no. CQ 0982) was used to assess cognitive coping strategies employed after negative or stressful events [30,31]. The adult CERQ is a 36-item self-report instrument comprising nine four-item subscales: Self-Blame, Acceptance, Rumination, Positive Refocusing, Refocus on Planning, Positive Reappraisal, Putting into Perspective, Catastrophizing, and Blaming Others. Items are rated on a five-point scale from 1 (“almost never”) to 5 (“almost always”), with higher scores indicating more frequent use of the respective cognitive emotion regulation strategy. In the present study, CERQ subscale scores were expressed as item-mean scores on the original 1–5 response metric and were interpreted dimensionally, not as diagnostic cut-offs. Consistent with the CERQ framework, Acceptance, Positive Refocusing, Refocus on Planning, Positive Reappraisal, and Putting into Perspective were treated as more adaptive cognitive coping strategies, whereas Self-Blame, Rumination, Catastrophizing, and Blaming Others were treated as less adaptive strategies.

In the adult psychometric study by Garnefski and Kraaij, the CERQ showed good factorial validity and internal consistency, with Cronbach’s alpha coefficients ranging from 0.75 to 0.87; one-year test–retest correlations ranged from 0.48 to 0.65, which the authors interpreted as adequate to good given the long retest interval [30].

The Quality of Life Inventory (QOLI; license no. MN-0079-00017402) was used to assess subjective quality of life and overall life satisfaction [32]. The QOLI is a 32-item self-report instrument covering 16 life domains: health, self-esteem, goals and values, money, work, play, learning, creativity, helping, love, friends, children, relatives, home, neighborhood, and community. For each domain, participants rate both the importance of that domain and their satisfaction with it, allowing the instrument to estimate quality of life as satisfaction in personally valued areas rather than as a simple sum of life-domain ratings. According to the scoring procedure, satisfaction ratings are weighted by domain importance (e.g., if a participant reports being very dissatisfied with the Money domain—satisfaction = −3—but rates this domain as not important—importance = 0, the weighted domain score is 0 (−3 × 0 = 0), meaning that this dissatisfaction does not reduce the global QOLI score. By contrast, the same level of dissatisfaction in a domain rated as very important would receive a strongly negative weighted score). The resulting global quality-of-life score is expressed as a T-score, with higher scores indicating better perceived quality of life. In the present study, the QOLI was used dimensionally to characterize perceived quality of life and to explore its associations with gambling symptom burden, personality dimensions, and cognitive emotion regulation strategies. The Quality of Life Inventory (QOLI) manual reports adequate reliability for the scale. Temporal stability of the QOLI T-scores was supported by a test–retest reliability coefficient of *r* = 0.73 over an approximately two-week interval (*N* = 55; mean interval = 14.4 days, SD = 3.9; *p* < 0.001). Internal consistency was also acceptable, with Cronbach’s α = 0.79, calculated for the summed weighted satisfaction scores across the 16 life domains. The manual specifies that this approach was used because the raw QOLI score is computed only from domains rated as important or very important, resulting in a variable number of contributing domains across respondents; nevertheless, the summed weighted satisfaction score showed an almost perfect association with the raw QOLI score (*r* = 0.99), supporting its adequacy as a substitute for internal-consistency estimation. In addition, the current version showed satisfactory comparability with the original QOLI version (*r* = 0.79, *p* < 0.001; *N* = 51). Taken together, these manual-based psychometric data support the reliability of the QOLI for assessing subjective quality of life/life satisfaction in the present study [33].

### 2.4. Administration and Scoring

All psychological assessments were administered individually by the principal investigator, a psychiatrist-researcher trained and licensed to use the assessment instruments. The questionnaires were completed either in a paper-and-pencil format or electronically via Google Forms. The same item wording, response options, and administration instructions were retained across both administration modalities. The use of Google Forms was limited to questionnaire delivery and response collection; scoring and interpretation were performed separately, according to the manuals and licensed scoring procedures of each instrument. All participants were Romanian-speaking clinical participants, and the instruments were used for research-based clinical characterization rather than for establishing stand-alone diagnoses.

Questionnaire responses were first entered into a Microsoft Excel database. For the PCF, item responses were subsequently entered into the licensed PCF scoring software provided by the test author/publisher. The software generated individual personality profiles, validity indicators, raw scores, normalized T-scores, and calibrated T-scores. In the present study, PCF results were reported as calibrated T-scores. PCF protocol validity was evaluated using the instrument’s validity indicators; 13 of the 122 PCF protocols did not meet the validity criteria and were excluded from PCF-based analyses, resulting in a valid PCF subsample of 109 participants. 

The CERQ was scored according to the authors’ instructions by aggregating the items corresponding to each of the nine cognitive emotion regulation strategies; for statistical reporting, subscale scores were expressed as item-mean scores on the original 1–5 response metric. 

The QOLI was scored according to the manual by weighting satisfaction ratings by the importance assigned to each life domain; the resulting global quality-of-life score was expressed as a T-score. Final scale scores were then transferred to the statistical dataset and analyzed.

### 2.5. Score Interpretation

PCF scores were interpreted using the calibrated T-score metric generated by the licensed scoring software. According to the PCF interpretive framework, T-scores range from 20 to 80. For clinical scales, scores between 20 and 59 are generally interpreted as having low or no clinical relevance, scores between 60 and 64 as borderline or possibly clinically relevant, scores between 65 and 69 as probably clinically relevant, and scores between 70 and 80 as highly clinically relevant. Higher scores indicate a stronger expression of the assessed dysfunctional trait, maladaptive personality domain, psychopathological tendency, personality-functioning impairment, or personality-disorder feature. For PCF validity indicators, T-score interpretation depends on the specific validity scale; elevated values may indicate moderate, marked, or extreme response distortion, and in some validity scales very low scores may also signal a distorted response style. Therefore, PCF validity indicators were used only to determine whether protocols could be interpreted and whether calibrated T-scores should be used.

CERQ scores were interpreted as indicators of the frequency with which participants used specific cognitive emotion regulation strategies after negative or stressful events. The full CERQ comprises nine subscales: Self-Blame, Acceptance, Rumination, Positive Refocusing, Refocus on Planning, Positive Reappraisal, Putting into Perspective, Catastrophizing, and Blaming Others. Each subscale contains four items scored from 1 (“almost never”) to 5 (“almost always”). In the present study, subscale scores were expressed as item-mean scores ranging from 1 to 5, with higher scores indicating more frequent use of the respective strategy. The five strategies Acceptance, Positive Refocusing, Refocus on Planning, Positive Reappraisal, and Putting into Perspective were treated as adaptive or more functional cognitive coping strategies, whereas Self-Blame, Rumination, Catastrophizing, and Blaming Others were treated as less adaptive cognitive coping strategies. CERQ scores were interpreted dimensionally and descriptively; they were not used as diagnostic cut-offs.

QOLI scores were interpreted as indicators of overall life satisfaction and perceived quality of life. QOLI scoring combines the importance assigned to each life domain with the participant’s satisfaction in that domain; weighted satisfaction ratings for individual domains range from -6 to +6, where negative values reflect dissatisfaction in personally important domains and positive values reflect satisfaction or fulfillment. The global QOLI score is expressed as a T-score, with higher scores indicating better perceived quality of life. Published QOLI interpretive ranges classify T-scores of 0–36 as very low quality of life, 37–42 as low, 43–57 as average, and 58–77 as high. These ranges were used to aid clinical interpretation of the descriptive findings.

### 2.6. Statistical Analysis

Statistical analyses were conducted using Microsoft Excel Version 16.96 and JASP (v. 0.96 Apple Silicon, University of Amsterdam). Prior to inferential analyses, the Shapiro–Wilk test confirmed significant departure from normality for the severity score (W = 0.481, *p* < 0.001), attributable to the pronounced ceiling effect. Accordingly, nonparametric methods were used throughout: Spearman’s rank-order correlation (ρ) for associations between variables; Mann–Whitney U for two-group comparisons; and Kruskal–Wallis H for comparisons across three or more groups. All tests were two-tailed with α = 0.05. Group descriptive statistics are reported as median and interquartile range (IQR). Ninety-five percent confidence intervals for Spearman’s ρ were computed using the Fisher z-transformation.

Given the large number of simultaneous Spearman correlations, p-values were adjusted using the Benjamini–Hochberg (BH) false discovery rate procedure, applied separately within four conceptually defined comparison families: (1) DSM severity ~ PCF scales (50 tests); (2) DSM severity ~ CERQ subscales (9 tests); (3) QOLI ~ PCF scales (50 tests); (4) QOLI ~ CERQ subscales (9 tests). Adjusted q-values and raw p-values are reported in all correlation tables. Correlations between DSM severity and QOLI (single test) and all demographic group comparisons were not subject to FDR correction.

For significant Kruskal–Wallis tests (*p* < 0.05), pairwise post hoc comparisons were conducted using Dunn’s test with Bonferroni correction. Marital status (4 groups, 6 pairs) and social support (3 groups, 3 pairs) yielded significant overall tests; post hoc results are reported in Tables S7 and S8. For occupational status (6 groups, 15 pairs), which reached overall significance (*p* = 0.020), Dunn post hoc results are reported in Table S9; no individual pair survived Bonferroni correction, indicating that the omnibus difference does not localize to a specific group contrast at the corrected threshold.

The study was based on an available clinical sample; no a priori sample size calculation was performed. Post hoc power estimates for *N* = 122, α = 0.05 (two-tailed) indicate ≥ 80% power to detect correlations of |ρ| ≥ 0.25, and ≥ 95% power for |ρ| ≥ 0.31. However, the restricted variance of the DSM score (ceiling effect) reduces the effective power of correlation analyses with this variable; observed effect sizes are, therefore, likely to be conservative underestimates of the true population associations. Small subgroups (widowed *N* = 3; high social support *N* = 10) are flagged accordingly. The sample size was sufficient to detect moderate associations but may have been underpowered for small effects, subgroup analyses, and multivariable models, especially after correction for multiple testing.

## 3. Results

### 3.1. Participant Characteristics, Gambling Behavior, and DSM-5-TR Severity Distribution

The study sample comprised 122 treatment-seeking participants, predominantly male (*n* = 107, 87.7%; female: *n* = 15, 12.3%). Most participants were aged 25–44 years (*n* = 79, 64.7%), had secondary/high-school or university-level education (*n* = 90, 73.7%), were employed full-time (*n* = 62, 50.8%), lived in urban areas (*n* = 81, 66.4%), and reported monthly incomes between 2000 and 5999 RON (*n* = 71, 58.2%). Participants were most commonly single (*n* = 47, 38.5%) or married/partnered (*n* = 42, 34.4%), while 30 were divorced or separated (24.6%). A family history of psychiatric disorders or addictive behaviors was reported by 76 participants (62.3%). Perceived social support was predominantly moderate (*n* = 68, 55.7%) or low (*n* = 44, 36.1%), with only 10 participants reporting high support (8.2%).

Gambling behavior was characterized by early onset, frequent participation, and prolonged sessions. Most participants initiated gambling before age 18 (*n* = 82, 67.2%), and almost all before age 25 (*n* = 115, 94.2%). The most reported gambling activities were sports betting (*n* = 88, 72.1%), online card games/poker (*n* = 80, 65.6%), and slot machines (*n* = 70, 57.4%). Overall, 107 participants (87.6%) gambled at least weekly, including 53 (43.4%) who gambled three or more times per week and 22 (18.0%) who gambled daily. Session duration was also substantial, with 90 participants (73.7%) reporting sessions lasting ≥3 h, most commonly 3–4 h (*n* = 48, 39.3%) or ≥5 h (*n* = 42, 34.4%).

The binary DSM-5-TR symptom burden index showed a pronounced ceiling effect (Mdn = 9, IQR = 9–9, range = 4–9; Shapiro–Wilk W = 0.481, *p <* 0.001). Most participants met all nine DSM-5-TR criteria (*n* = 95, 77.9%), followed by eight criteria (*n* = 16, 13.1%), seven criteria (*n* = 8, 6.6%), and five or fewer criteria (*n* = 3, 2.5%). Accordingly, 111 participants (91.0%) were classified as severe, 8 (6.6%) as moderate, and 3 (2.5%) as mild; no participant fell below the diagnostic threshold of four criteria. The need to gamble with increasing amounts of money was endorsed by all participants (100.0%), followed by persistent preoccupation with gambling and chasing losses, each endorsed by 99.2% of participants. Lying about gambling involvement was present in 98.4% of cases, while restlessness or irritability when attempting to stop, gambling to escape dysphoric mood, and jeopardized relationships or opportunities were each endorsed by 96.7% of participants. Repeated unsuccessful attempts to stop gambling were reported by 94.3% of participants, whereas reliance on others for money was the least frequently endorsed criterion, although it was still present in 81.1% of the sample. This distribution is consistent with recruitment from a specialized inpatient and day-hospital psychiatric setting serving treatment-seeking patients with established, clinically significant gambling disorder. The restricted score variance should therefore be considered when interpreting subsequent correlation analyses and is further addressed in the Limitations section.

### 3.2. Descriptive Statistics of PCF, CERQ, and QOLI Measures

The PCF was used to perform a multidimensional assessment of participants’ personality traits. Of the total sample of 122 participants, 13 produced an invalid result on the instrument’s validity scale and were, therefore, excluded from PCF-based analyses; consequently, PCF analyses were conducted in a subsample of *N* = 109 participants, whereas CERQ and QOLI analyses were conducted in the full sample (*N* = 122). Consistent with the nonparametric analytic approach, central tendency and dispersion are reported as medians and interquartile ranges (IQR); corresponding means and standard deviations are provided in Supplementary Table S4.

PCF scores are expressed as calibrated T-scores (M = 50, SD = 10 in the normative sample), with higher values indicating a more pronounced expression of the assessed dimension. In the present clinical subsample, the medians of most PCF scales fell within the borderline to clinically relevant range (T ≈ 60–69), reflecting an elevated overall personality-pathology profile. Full scale-level descriptive statistics are reported in Supplementary Table S4. 

Thirteen participants produced invalid PCF profiles and were excluded from PCF-based analyses only. The PCF-invalid subgroup represented 10.7% of the total sample and was predominantly male (92.3%), aged 35–44 years (53.8%), and more often from rural areas (69.2%). Low perceived social support was reported by 61.5% of these participants. Compared with participants with valid PCF profiles, PCF-invalid participants did not differ significantly in DSM-related symptom burden or QOLI T-score. However, descriptive comparisons suggested that invalid PCF profiles were more frequent among rural participants, participants without occupation, and those with lower educational attainment. Given the small size of the invalid-profile subgroup, these findings should be interpreted cautiously and reported as a potential selection issue rather than as definitive group differences. Review of the PCF validity indicators showed that invalidity was primarily driven by elevated Inconsistent Responses (IC): 12 of the 13 invalid protocols showed extreme inconsistency or random responding. Four protocols also showed elevated Infrequent Responses (IF), indicating an atypical response pattern in addition to inconsistency. One protocol was invalidated due to extremely low Self-disclosure Amplitude (DA), suggesting marked underreporting or insufficient endorsement of clinical symptoms, while one protocol showed a mixed invalidity pattern involving IC, IF, DA, and Desirable Responses (DS). Thus, the invalid PCF profiles reflected instrument-specific response-validity problems rather than participant exclusion from the study. These participants remained eligible for all non-PCF analyses, including DSM-5 gambling-disorder criteria, CERQ, QOLI, and sociodemographic variables, when complete data were available.

Among the maladaptive CERQ strategies, Blaming Others showed the highest median (Mdn = 3.00, IQR 2.25–3.75), followed by Self-Blame (Mdn = 2.75, IQR 2.25–3.25), Rumination (Mdn = 2.50, IQR 2.25–3.25), and Catastrophizing (Mdn = 2.25, IQR 1.75–3.00).

Among the adaptive strategies, the highest medians were observed for Refocus on Planning (Mdn = 3.25, IQR 2.75–4.00) and Acceptance (Mdn = 3.25, IQR 2.75–3.75), indicating that participants relied predominantly on these two strategies. Lower medians were recorded for Putting into Perspective (Mdn = 2.50, IQR 2.00–3.00), Positive Reappraisal (Mdn = 2.25, IQR 1.75–3.00), and Positive Refocusing (Mdn = 2.00, IQR 1.50–3.00). The comparatively limited use of Positive Reappraisal and Positive Refocusing suggests a reduced tendency to cognitively restructure negative situations in a constructive manner.

Quality of life, indexed by the QOLI global T-score, was very low overall (Mdn = 35.00, IQR 29.00–42.00; range 20–66). Using published QOLI interpretive ranges, 67 participants (54.9%) scored in the very low range, 27 (22.1%) in the low range, 25 (20.5%) in the average range, and 3 (2.5%) in the high range. The sample median fell within the very low range, indicating markedly reduced perceived quality of life in this treatment-seeking sample. Detailed descriptive statistics for all CERQ subscales, the QOLI global score, and all PCF scales are provided in Supplementary Table S4.

### 3.3. Associations Between PCF Scores and DSM Symptom Severity

Spearman correlations between the binary DSM symptom burden index and PCF scales (*N* = 109; 50-test FDR family) are presented in Table S2. Despite the restricted variance of the severity score, 47 of 50 PCF indicators showed significant positive associations after BH correction, indicating robust associations with the available variability in the sample.

Among psychopathological tendency scales, all three were significantly positively associated with severity: Behavioral Problems (ρ = 0.388, 95% CI [0.216, 0.537], *q* < 0.001), Emotional Problems (ρ = 0.349 [0.172, 0.504], *q* = 0.001), and Cognitive Problems (ρ = 0.338 [0.161, 0.495], *q* = 0.001). All five maladaptive personality dimensions were significant: Negative Emotionality (ρ = 0.369), Psychoticism (ρ = 0.305), Antagonism (ρ = 0.323), Disinhibition (ρ = 0.227), and Detachment (ρ = 0.297). Among personality disorder scales, all 10 were significant after FDR correction, with the strongest association for Borderline PD (ρ = 0.408 [0.238, 0.553]). All six personality functioning indicators were significant (ρ = 0.318–0.430). The only non-significant traits were Grandiosity (ρ = 0.051, *q* = 0.597), Risk Taking (ρ = 0.167, *q* = 0.086), and Rigid Perfectionism (ρ = 0.152, *q* = 0.116).

### 3.4. Associations Between CERQ Scores and DSM Symptom Severity

Spearman correlations between the DSM symptom burden index and CERQ subscales (*N* = 122; 9-test FDR family) are presented in Supplementary Table S3. Among maladaptive strategies, Catastrophizing (ρ = 0.289 [0.117, 0.444], *q* = 0.007), and Blaming Others (ρ = 0.284 [0.112, 0.440], *q* = 0.007) were significantly positively associated with severity. Rumination and Self-Blame were not significant after correction.

Among adaptive strategies, three subscales showed significant negative associations with severity after FDR correction: Positive Reappraisal (ρ = −0.268 [−0.426, −0.095], *q* = 0.007), Putting into Perspective (ρ = −0.267 [−0.425, −0.094], *q* = 0.007), and Positive Refocusing (ρ = −0.205 [−0.369, −0.028], *q* = 0.042). These findings indicate that greater use of adaptive reappraisal-based strategies is associated with lower symptom severity. Acceptance and Refocus on Planning were not significant.

### 3.5. Associations Between QOLI and PCF Scores

Quality of life associations with PCF scales (*N* = 109; 50-test FDR family) are presented in Table S4. Among psychopathological tendencies, Emotional Problems (ρ = −0.325, *q* = 0.005) and Cognitive Problems (ρ = −0.235, *q* = 0.049) were significant; Behavioral Problems was not. Significant trait-level associations (all *q* ≤ 0.05) included Submissiveness (ρ = −0.366), Eccentricity (ρ = −0.343), Diffuse Identity (ρ = −0.346), Problematic Self (ρ = −0.330), Depression (ρ = −0.287), Negative Emotionality (ρ = −0.284), Shyness (ρ = −0.264), Perseveration (ρ = −0.248), and Anhedonia (ρ = −0.243). Among personality disorders, only Borderline (ρ = −0.337) and Dependent (ρ = −0.273) were significant. Among personality functioning indicators, Diffuse Identity, Problematic Self, and Impaired Goals were significant.

### 3.6. Correlations Between CERQ Scores and Quality of Life (QOLI)

Among CERQ subscales (*N* = 122; 9-test FDR family; Table S5), only Refocus on Planning showed a significant positive association with quality of life (ρ = 0.285 [0.113, 0.440], *q* = 0.008), and only Catastrophizing showed a significant negative association (ρ = −0.281 [−0.437, −0.109], *q* = 0.008). No other subscale reached significance after BH correction.

### 3.7. Association Between DSM Severity and Quality of Life

The direct correlation between the binary DSM symptom burden index and QOLI T-score was not statistically significant (ρ = −0.100, 95% CI [−0.273, 0.079], *p* = 0.273, *N* = 122). Given the pronounced ceiling effect and restricted variability of the DSM-5-TR criterion count, this non-significant result should not be interpreted as evidence that gambling-related symptom burden is unrelated to quality of life across the full continuum of gambling disorder severity. Rather, it indicates that no statistically significant association was detected within this highly severe treatment-seeking sample.

### 3.8. Correlations Between Symptom Severity and Demographic Data

Results of nonparametric comparisons are presented in Table S6. Given the pronounced ceiling effect of the symptom burden index (Mdn = 9 for most subgroups), differences in medians are minimal; comparisons are primarily driven by differences in rank distributions. Results should be interpreted accordingly.

Post hoc -Marital Status (H (3) = 8.756, *p* = 0.033). Dunn’s test (6 pairs, Bonferroni corrected) identified one significant pairwise difference: Single participants had significantly higher severity ranks than married/partnered participants (*z* = 2.930, p_bonf = 0.020). No other pair was significant after correction (Table S7). 

Post hoc -Social Support (H (2) = 11.157, *p* = 0.004). Dunn’s test (3 pairs, Bonferroni corrected) identified two significant differences: both Low support and Moderate support participants had significantly higher severity than High support participants (Low vs. High: *z* = 3.340, p_bonf = 0.003; Moderate vs. High: *z* = 2.849, p_bonf = 0.013). The Low vs. Moderate comparison was not significant (p_bonf = 0.868; Table S8). Note the small High support group (*n* = 10). 

Post hoc -Occupational Status (H(5) = 13.433, *p* = 0.020). Dunn’s test (15 pairs, Bonferroni corrected) did not identify any significant pairwise difference after correction. The omnibus test significance thus reflects a general distributional difference across occupational categories rather than a specific group contrast. Full-time employed participants showed a lower rank distribution compared to part-time and other groups, but no pair survived correction (Table S9).

### 3.9. Synthesis of Results in Relation to the Study Objectives and Hypotheses

This study examined the associations between DSM-related gambling symptom burden, personality dimensions, cognitive emotion regulation strategies, quality of life, and sociodemographic variables. The extent to which each hypothesis was statistically supported is summarized in Table S10. H1 was supported: symptom burden was positively associated with all PCF psychopathological tendencies, all five maladaptive personality dimensions, the great majority of maladaptive traits, all ten personality-disorder feature scales, and all six personality-functioning indicators after Benjamini–Hochberg correction. H2 and H3 were partially supported, reflecting selective rather than uniform associations with cognitive emotion regulation strategies and with quality of life. The direct association between symptom burden and quality of life was not statistically significant. H4 was partially supported: symptom burden differed significantly by marital status and perceived social support, showed an unlocalized omnibus difference by occupational status, and did not differ by sex, age, education, income, residence, or family history.

## 4. Discussion

### 4.1. Main Findings 

The present exploratory study provides a multidimensional characterization of gambling disorder severity in a Romanian treatment-seeking psychiatric sample. Overall, the findings indicate that DSM-5 symptom burden was most consistently associated with maladaptive personality characteristics and selected cognitive emotion regulation strategies, whereas quality of life was more closely related to broader personality and coping dimensions than to the binary DSM-based symptom burden index itself. 

The sociodemographic profile of the sample was broadly consistent with established epidemiological and clinical patterns in gambling disorder. Participants were predominantly male, and most were concentrated in early and middle adulthood, particularly within the 25–34 and 35–44 age groups. This aligns with systematic-review evidence indicating that being male, young, single, socially isolated, and financially vulnerable are among the most frequently reported risk correlates for gambling disorder. In a systematic review of risk factors for gambling disorder, Moreira et al. identified being a single young male, living alone, lower educational attainment, and financial difficulties as relevant risk factors for developing or maintaining gambling-related problems [15]. In the present study, however, these sociodemographic findings should be interpreted as characteristics of a specialized clinical sample rather than as population-level risk estimates.

The gambling-behavior profile further supports the clinical severity of the sample. Most participants reported early onset of gambling, frequent gambling participation, and prolonged sessions. The most frequently reported activities were sports betting, online card games/poker, and slot machines. This pattern is compatible with recent literature emphasizing the role of highly accessible and rapidly reinforcing gambling formats. The World Health Organization notes that betting, slot machines, casino games, lotteries, and bingo are common forms of gambling, and that electronic gambling machines and casino games are often associated with higher risk of harm, both in physical and online environments [2]. The high prevalence of sports betting in the present sample is also consistent with recent longitudinal evidence in young men showing that sports gambling frequency is a salient predictor of later problem-gambling severity [34].

The binary DSM-5-TR symptom burden index showed a pronounced ceiling effect, with 91.0% of participants meeting criteria for severe gambling disorder and 77.9% meeting all nine DSM-5-TR criteria. This distribution is expected in a specialized inpatient and day-hospital psychiatric sample, but it has important implications for interpretation. Because most participants clustered at the upper end of the severity range, the score had limited variability, which may have attenuated correlations with psychological and quality-of-life variables. Therefore, significant associations observed despite this restriction are notable exploratory findings, whereas non-significant associations, particularly with quality of life, should not be interpreted as definitive evidence of absence of relationship.

Personality dimensions assessed by the PCF showed the most consistent associations with DSM-related gambling symptom burden. All three psychopathological tendency scales, all five maladaptive personality dimensions, all six personality-functioning indicators, and all ten personality-disorder feature scales were significantly associated with symptom severity after correction for multiple comparisons. This broad pattern suggests that the DSM-5-TR criterion count was associated with several personality-related dimensions in this treatment-seeking sample. However, these associations should not be interpreted as a personality profile specific to gambling disorder, because they may also reflect general psychopathological burden, negative affectivity, distress, comorbid conditions, or content overlap between clinical constructs. The strongest personality-disorder association reported in the Results section was observed for borderline personality features, which is consistent with literature linking gambling problems to emotional instability, impulsivity, interpersonal dysfunction, and broader personality pathology. A recent systematic review of personality disorders in gambling disorder emphasized that impulsivity, emotional instability, sensation seeking, and interpersonal dysfunction have repeatedly been linked to problematic gambling behavior [17].

The dimensional personality findings are also consistent with broader trait-based evidence. Meta-analytic data on the five-factor model indicate that problem gambling is most strongly associated with higher neuroticism and lower conscientiousness and agreeableness. Dudfield et al. reported neuroticism as the strongest personality correlate of problem gambling, followed by negative associations with conscientiousness, agreeableness, openness, and extraversion [35]. Although the PCF framework is not identical to the five-factor model, the present findings involving Negative Emotionality, Antagonism, Disinhibition, Detachment, and Psychoticism are conceptually compatible with a broader pattern of personality-related dimensions associated with DSM-5-TR criterion count in this clinical sample, including emotional dysregulation, interpersonal difficulties, impulsivity, and maladaptive self-other functioning. At the same time, because the present study did not systematically assess comorbid mood, anxiety, substance-use, ADHD, or suicidality diagnoses, these associations should not be interpreted as specific to gambling disorder alone. A recent narrative review emphasizes that gambling disorder is highly comorbid with substance and alcohol use disorders, mood disorders, and anxiety disorders, supporting the need to account for general psychopathological burden in future multivariable analyses [36]. 

Cognitive emotion regulation strategies were associated with gambling severity in a more selective pattern. Among maladaptive strategies, Catastrophizing and Blaming Others were positively associated with DSM-related severity, whereas Rumination and Self-Blame did not remain significant after correction. Among strategies usually considered adaptive, Positive Reappraisal, Putting into Perspective, and Positive Refocusing were negatively associated with severity, while Acceptance and Refocus on Planning were not significant. This pattern partly converges with previous literature showing that emotion dysregulation is relevant to gambling disorder but also highlights that not all cognitive strategies relate to gambling severity in the same way. A systematic review of ERQ/CERQ studies in adults with gambling disorder found consistent evidence for reduced cognitive reappraisal, greater expressive suppression, and greater use of maladaptive cognitive strategies such as rumination, catastrophizing, and self-blame [21].

The negative associations observed for Positive Reappraisal, Putting into Perspective, and Positive Refocusing suggest that greater use of constructive cognitive reframing may be linked to lower symptom burden in this clinical sample. However, this interpretation should remain cautious. Prior research has shown that strategies generally considered adaptive may function differently in gambling contexts. Navas et al. found that gambling disorder patients used Self-Blame and Catastrophizing more than controls but also used Positive Refocusing more often; moreover, putatively adaptive CERQ strategies shared variance with gambling severity and gambling-related cognitions [22]. Thus, adaptive emotion regulation strategies may reduce symptom burden in some contexts but may also support biased reinterpretations of gambling outcomes in others. The present cross-sectional findings therefore support an association, not a causal or protective effect. 

Because the sample was recruited in a treatment-seeking psychiatric setting, the PCF, CERQ, and QOLI findings may have been influenced by unmeasured comorbid psychiatric symptoms, medication status, psychotherapy status, treatment phase, or acute distress at assessment. Therefore, the observed associations should be interpreted as exploratory clinical associations rather than as disorder-specific correlates of gambling disorder.

Quality of life was very low overall in the sample, with most participants scoring in the low or very low QOLI range. However, the direct association between the binary DSM-5-TR symptom burden index and QOLI T-score was not statistically significant. This finding should be interpreted in light of the restricted variance of the symptom burden index and the high clinical severity of the sample. It is also consistent with emerging evidence suggesting that well-being among gamblers may be shaped substantially by co-occurring psychopathology rather than by gambling-problem severity alone. In a large population-based study of gamblers, Martí Valls et al. found that gambling problems were associated with lower well-being, but when internalizing, thought-disorder, externalizing, and obsessive-compulsive symptoms were modeled together, internalizing, thought-disorder, and externalizing symptoms remained significant predictors of lower well-being, while gambling problems no longer did [25]. The present study similarly suggests that subjective quality of life in gambling disorder may be more closely related to emotional problems, cognitive problems, negative emotionality, identity disturbance, depressive traits, and coping style than to the number of DSM criteria met.

The associations between QOLI and personality variables further support this interpretation. Lower quality of life was associated with Emotional Problems, Cognitive Problems, Negative Emotionality, several maladaptive traits, borderline and dependent personality features, and selected indicators of personality-functioning impairment. Among CERQ strategies, Catastrophizing was negatively associated with quality of life, whereas Refocus on Planning was positively associated. Taken together, these findings suggest that perceived quality of life in gambling disorder may depend on broader emotional, cognitive, interpersonal, and self-regulatory functioning. Future studies should examine whether personality dysfunction and cognitive emotion regulation mediate or moderate the relationship between gambling severity and quality of life.

Among sociodemographic and contextual variables, marital status and perceived social support showed the clearest associations with DSM-related symptom burden. Single participants had significantly higher severity ranks than married or partnered participants, and participants reporting low or moderate social support had higher severity than those reporting high support. These findings are consistent with longitudinal registry evidence indicating that marital status is a robust correlate of gambling disorder, that divorce is associated with higher odds of subsequent gambling disorder diagnosis, and that transition into marriage is associated with lower odds of gambling disorder diagnosis [37]. They are also consistent with treatment-outcome research showing that low baseline social support is associated with greater gambling severity, family and psychiatric problems, and poorer post-treatment outcomes [38]. In the present sample, occupational status also showed a significant omnibus association with severity, but no individual post hoc contrast survived Bonferroni correction; this result should therefore be interpreted as an unlocalized distributional difference rather than as evidence that one occupational subgroup had definitively higher severity.

In summary, the present findings support a multidimensional clinical understanding of gambling disorder. DSM-related symptom burden co-occurred with a broader pattern of maladaptive personality functioning, emotion regulation patterns, and relational-contextual vulnerability in this treatment-seeking sample. The results are consistent with previous literature emphasizing the roles of negative emotionality, personality pathology, emotional dysregulation, social support, and comorbid psychopathology in gambling disorder. However, because of the cross-sectional design, the absence of systematic comorbidity assessment, and the pronounced ceiling effect of the binary DSM symptom burden index, these findings should be interpreted as exploratory and hypothesis-generating rather than causal or disorder-specific.

### 4.2. Limitations

The interpretation of the present findings should be considered in light of several methodological limitations that collectively define the scope and generalizability of the conclusions.

The cross-sectional design permits identification of statistical associations but precludes causal or directional inference. Whether maladaptive personality traits constitute predisposing vulnerability factors for more severe gambling behavior, or whether chronic gambling exacerbates pre-existing personality features, cannot be determined from the present data. Longitudinal designs are needed to clarify temporal ordering.

The binary DSM-5 symptom burden index showed a pronounced ceiling effect in this clinical sample: 91.0% of participants were classified as severe (8–9 criteria met), with Mdn = 9 and IQR = 9–9 for most subgroups. This restricted variance reduces the statistical power of correlational analyses and attenuates effect size estimates; the reported associations are therefore likely conservative underestimates of the true population relationships. This ceiling effect is an inherent consequence of using a diagnostic binary threshold score (present/absent) in a treatment-seeking clinical population where all participants meet the disorder criteria and most present with severe symptomatology. Future research in less severely affected or community samples would benefit from continuous dimensional measures of gambling-related severity validated against population norms, such as the Problem Gambling Severity Index (PGSI) or the Gambling Symptom Assessment Scale (G-SAS). The nine-item DSM-related symptom burden score was locally operationalized for this study and has not been externally validated against established gambling-severity instruments such as the PGSI, G-SAS, or SOGS. It should, therefore, be regarded as a pragmatic, study-specific index rather than a validated severity scale.

This study did not include systematic assessment of co-occurring psychiatric conditions. No structured diagnostic interview for comorbid disorders -such as major depressive disorder, anxiety disorders, bipolar spectrum conditions, substance use disorders, ADHD, or suicidality nor medication status, psychotherapy status, or treatment history, was administered. Rates of co-occurring mood, anxiety, and substance use disorders in clinical GD samples frequently exceed 50–70%. Without controlling for these conditions, it cannot be determined whether the reported associations between personality dimensions, emotion regulation strategies, and GD severity are specific to gambling disorder or partially reflect a general psychopathological burden. Future studies should incorporate validated diagnostic instruments (e.g., MINI International Neuropsychiatric Interview) and employ multivariable models to disentangle independent contributions of GD severity from comorbid psychopathology. 

The analyses relied on bivariate Spearman correlations and nonparametric group comparisons without multivariable modeling. This approach does not allow estimation of the independent contribution of each predictor domain, nor the assessment of mediation or moderation pathways. The large number of PCF scales within each FDR family introduces substantial multicollinearity, meaning individual coefficients reflect shared variance rather than unique explanatory power. Future studies should employ dimension-reduction approaches or regularized regression to address multicollinearity.

The sample was predominantly male (87.7%) and recruited from a single specialized psychiatric center, limiting generalizability to community, primary care, or online gambling populations. Subgroups were unequal in size; the widowed group (*N* = 3) and high social support group (*N* = 10) are too small for reliable nonparametric inference. The combination of retrospective identification and prospective enrolment may also have introduced heterogeneity in the interval between initial diagnosis and research assessment.

All three psychological instruments (PCF, CERQ, QOLI) are self-report measures susceptible to social desirability, limited self-insight, and mood-congruent bias. Thirteen participants (10.7%) produced invalid PCF profiles and were excluded from personality-based analyses. No test–retest data are available. The exclusion of 13 invalid PCF profiles, although methodologically warranted, may introduce a degree of selection bias into the personality-related analyses. In addition, assessments were completed either on paper or via an identical Google Forms version; although item wording, response options, and instructions were identical across paper-and-pencil and Google Forms administration, mode-of-administration effects cannot be excluded. Completion in a hospital setting in the presence of the researcher may differ from remote electronic completion in terms of privacy, perceived anonymity, environmental distractions, and willingness to disclose clinically sensitive information.

Without a healthy or clinical comparison group, it is not possible to determine whether the observed personality profiles and emotion regulation patterns are specific to gambling disorder or represent non-specific correlates of treatment-seeking behavior or general psychopathology.

Taken together, these limitations situate the present study as a preliminary, hypothesis-generating, cross-sectional characterization of psychological profiles associated with gambling disorder severity in a Romanian specialized clinical sample. The findings should not be interpreted as causal, disorder-specific, or generalizable beyond similar clinical settings without replication in broader samples using validated dimensional measures.

## 5. Conclusions

In this exploratory cross-sectional treatment-seeking sample, the DSM-5-TR criterion count was associated with several personality-related and cognitive emotion regulation measures. Because the criterion count showed a pronounced ceiling effect and was used as a pragmatic symptom-burden index rather than as a validated dimensional severity scale, the findings should be interpreted as exploratory associations within a restricted clinical range. 

The absence of a statistically significant association between the criterion count and QOLI T-score should not be taken as evidence that gambling-related symptom burden is unrelated to quality of life across the full severity continuum. Rather, quality of life appeared more closely associated with selected personality-related and coping variables in this sample. These findings generate hypotheses for future research and require replication in larger, prospectively recruited samples with broader severity variability and systematic assessment of psychiatric comorbidity, medication use, psychotherapy status, and treatment history.

## Data Availability

The original contributions presented in this study are included in the article and Supplementary Materials. Further inquiries can be directed to the corresponding authors.

## Supplementary Materials

The following supporting information can be downloaded at: https://www.mdpi.com/article/10.3390/clinpract16070122/s1.

## Abbreviations

The following abbreviations are used in this manuscript:

DSM	Diagnostic and Statistical Manual of Mental Disorders
GD	Gambling Disorder
DSM-5	Diagnostic and Statistical Manual of Mental Disorders, Fifth Edition
PCF	Personality Clinical Form
CERQ	Cognitive Emotion Regulation Questionnaire
QOLI	Quality of Life Inventory
SOGS-RA	South Oaks Gambling Screen—Revised for Adolescents
20 GA-RA	20 Questions of Gamblers Anonymous—Revised for Adolescents
DSM-IV	Diagnostic and Statistical Manual of Mental Disorders, Fourth Edition
ICD-10	International Classification of Diseases, Tenth Revision
PGSI	Problem Gambling Severity Index
SCID-5-CV	Structured Clinical Interview for DSM-5 Disorders—Clinician Version
r_it Corrected	Item-Total Correlation
PID-5	Personality Inventory for DSM-5
SCID-5-PD-SPQ	Structured Clinical Interview for DSM-5 Personality Disorders—Self-Report Personality Questionnaire
OMNI-IV	OMNI Personality Inventory, Fourth Edition
MCMI-III	Millon Clinical Multiaxial Inventory, Third Edition
MMPI-2	Minnesota Multiphasic Personality Inventory, Second Edition
DAPP-BQ	Dimensional Assessment of Personality Pathology—Basic Questionnaire
LPFS-BF	Level of Personality Functioning Scale—Brief Form
RON	Romanian leu
IC	Inconsistent Responses
IF	Infrequent Responses
DA	Self-disclosure Amplitude
DS	Desirable Responses
CI	Confidence Interval
PD	Personality Disorder
ADHD	Attention-Deficit/Hyperactivity Disorder
ERQ	Emotion Regulation Questionnaire
G-SAS	Gambling Symptom Assessment Scale
SOGS	South Oaks Gambling Screen
MINI	Mini International Neuropsychiatric Interview
IX	Emotional Problems
EX	Behavioral Problems
TX	Cognitive Problems
NG	Negative Emotionality
DM	Social and Affective Detachment
AG	Antagonism
DH	Disinhibition
PT	Psychoticism
TPBO	Borderline Personality Disorder
ID	Diffuse Identity
SC	Impaired Goals
EM	Deficient Empathy
IT	Impaired Intimacy
SELF	Problematic Self
REL	Impaired Relationships
TPDP	Dependent Personality Disorder

## References

[1] Lucas I., Granero R., Fernández-Aranda F., Solé-Morata N., Demetrovics Z., Baenas I., Gómez-Peña M., Moragas L., Mora-Maltas B., Lara-Huallipe M.L. (2023). Gambling disorder duration and cognitive behavioural therapy outcome considering gambling preference and sex. J. Psychiatr. Res..

[2] World Health Organization (2024). Gambling [Internet].

[3] Tran L.T., Wardle H., Colledge-Frisby S., Taylor S., Lynch M., Rehm J., Volberg R., Marionneau V., Saxena S., Bunn C. (2024). The prevalence of gambling and problematic gambling: A systematic review and meta-analysis. Lancet Public Health.

[4] Rizeanu S., Vezeteu E. (2017). Incidence of problem gambling in Romania: Brief report. J. Psychol. Brain Stud..

[5] Lupu V., Lupu I.R. (2018). Romanian National Prevalence Study: Problem and pathological gambling in children and adolescents. Clujul Med..

[6] American Psychiatric Association (2022). Diagnostic and Statistical Manual of Mental Disorders.

[7] Petry N.M., Blanco C., Auriacombe M., Borges G., Bucholz K., Crowley T.J., Grant B.F., Hasin D.S., O’bRien C. (2014). An overview of and rationale for changes proposed for pathological gambling in DSM-5. J. Gambl. Stud..

[8] Cowlishaw S., Merkouris S.S., Dowling N.A., Rodda S., Suomi A., Thomas S.L. (2019). Locating gambling problems across a continuum of severity: Rasch analysis of the Quinte Longitudinal Study (QLS). Addict. Behav..

[9] Strong D.R., Kahler C.W. (2007). Evaluation of the continuum of gambling problems using the DSM-IV. Addiction.

[10] Ferris J., Wynne H. (2001). The Canadian Problem Gambling Index: Final Report.

[11] Holtgraves T. (2009). Evaluating the Problem Gambling Severity Index. J. Gambl. Stud..

[12] Currie S.R., Hodgins D.C., Casey D.M. (2013). Validity of the Problem Gambling Severity Index interpretive categories. J. Gambl. Stud..

[13] Williams R.J., Volberg R.A. (2014). The classification accuracy of four problem gambling assessment instruments in population research. Int. Gambl. Stud..

[14] Yau Y.H., Potenza M.N. (2015). Gambling disorder and other behavioral addictions: Recognition and treatment. Harv. Rev. Psychiatry.

[15] Moreira D., Azeredo A., Dias P. (2023). Risk factors for gambling disorder: A systematic review. J. Gambl. Stud..

[16] Mari E., Cricenti C., Boccia M., Zucchelli M.M., Nori R., Piccardi L., Giannini A.M., Quaglieri A. (2024). Betting on your feelings: The interplay between emotion and cognition in gambling affective task. J. Clin. Med..

[17] Ioniță I., Mușat M.I., Cătălin B., Ciobanu C.A., Ciobanu A.M. (2026). A systematic review of personality disorders in patients with gambling disorder. Clin. Pract..

[18] Müller K.W., Werthmann J., Beutel M.E., Wölfling K., Egloff B. (2021). Maladaptive personality traits and their interaction with outcome expectancies in gaming disorder and internet-related disorders. Int. J. Environ. Res. Public Health.

[19] Maniaci G., La Cascia C., Picone F., Lipari A., Cannizzaro C., La Barbera D. (2017). Predictors of early dropout in treatment for gambling disorder: The role of personality disorders and clinical syndromes. Psychiatry Res..

[20] Fierro I., Fernández-Prieto R., Fernández-Parra A., Herrero-Martín M., Herrero A.J. (2024). Personality traits and physical activity in patients with gambling disorder attending a rehabilitation center: An observational study. Front. Psychol..

[21] Ioniță I., Mușat M.I., Cătălin B., Ciobanu A.M. (2026). Assessment of cognitive emotion regulation in gambling disorder: A systematic review of the literature. Clin. Pract..

[22] Navas J.F., Verdejo-García A., López-Gómez M., Maldonado A., Perales J.C. (2016). Gambling with rose-tinted glasses on: Use of emotion-regulation strategies correlates with dysfunctional cognitions in gambling disorder patients. J. Behav. Addict..

[23] Ruiz de Lara C.M., Navas J.F., Perales J.C. (2019). The paradoxical relationship between emotion regulation and gambling-related cognitive biases. PLoS ONE.

[24] Tulloch C., Browne M., Hing N., Rockloff M., Hilbrecht M. (2023). How gambling harms others: The influence of relationship-type and closeness on harm, health, and wellbeing. J. Behav. Addict..

[25] Martí Valls C., Håkansson A., Cervin M. (2025). Understanding well-being among gamblers: The role of co-occurring psychopathology. Psychiatry Res..

[26] First M.B., Williams J.B.W., Karg R.S., Spitzer R.L. (2016). Structured Clinical Interview for DSM-5 Disorders—Clinician Version (SCID-5-CV).

[27] Sava F.A. (2020). Formularul Clinic de Personalitate (Personality Clinical Form—PCF): Manualul Utilizatorului Romanian.

[28] Sava F.A. (2020). Personality Clinical Form (PCF) [Internet].

[29] Sava F.A., Dindelegan C., Sălăgean N. (2020). The utility of the Personality Clinical Form indicators of response distortion: Receiver operating characteristic analysis. Rom. J. Appl. Psychol..

[30] Garnefski N., Kraaij V. (2007). The Cognitive Emotion Regulation Questionnaire. Eur. J. Psychol. Assess..

[31] Garnefski N., Kraaij V., Spinhoven P. (2010). CERQ: Manual de Utilizare a Chestionarului de Coping Cognitiv-Emoțional; Adaptarea și Standardizarea CERQ pe Populația din România.

[32] Frisch M.B., Maggino F. (2024). Quality-of-Life-Inventory. Encyclopedia of Quality of Life and Well-Being Research.

[33] Frisch M.B. (2025). QOLI—Quality of Life Inventory = Inventarul Calității Vieții: Manual și Ghid de Tratament; Romanian Adaptation by Livinți R.

[34] Mancini V.O., Brett J.D., Heirene R.M., Fisher K., Nevill T.P., Mitrou F. (2025). Predicting problem gambling in young men: The impact of sports gambling frequency and internalizing symptoms. J. Gambl. Stud..

[35] Dudfield F.W.H., Malouff J.M., Meynadier J. (2023). The association between the five-factor model of personality and problem gambling: A meta-analysis. J. Gambl. Stud..

[36] Sharma R., Weinstein A. (2025). Gambling disorder comorbidity: A narrative review. Dialogues Clin. Neurosci..

[37] Syvertsen A., Leino T., Pallesen S., Smith O.R.F., Sivertsen B., Griffiths M.D., Mentzoni R.A. (2023). Marital status and gambling disorder: A longitudinal study based on national registry data. BMC Psychiatry.

[38] Petry N.M., Weiss L. (2009). Social support is associated with gambling treatment outcomes in pathological gamblers. Am. J. Addict..

