# Supplementary material for: Exploratory Associations of Personality Traits, Cognitive Emotion Regulation, and Quality of Life with DSM-Related Symptom Burden in Gambling Disorder
Source: Clin Pract. 2026 Jun 29;16(7):122. doi: 10.3390/clinpract16070122 (PMC13407655; doi:10.3390/clinpract16070122)
Supplement: Supplementary file 1 [file clinpract-16-00122-s001.zip › Supplementary Materials S1.pdf]

Supplementary Materials S1 - Figure S1. Study Design and Analytic Workflow

Study design: Exploratory observational cross-sectional study

↓

Setting: 'Prof. Dr. Alexandru Obregia' Clinical Hospital of Psychiatry, Bucharest, Romania

↓

Sampling frame

Hospital database screening after ethics approval (Ethics Committee No. 35410/04.12.2025)

Adult patients admitted to inpatient or day-hospital services with ICD-10 F63.0 Pathological Gambling

Study period: 1 October 2023 – 31 January 2026

Potentially eligible cases: n = 251

↓

Recruitment

Retrospective identification of pre-approval cases and prospective recruitment of post-approval cases

Participant contact and enrolment began on 8 December 2025

No psychometric research assessment before ethics approval or informed consent

↓

Eligibility criteria

**Inclusion**	**Exclusion**
• Age ≥ 18 years<br>• Informed consent provided<br>• Capacity to understand study requirements<br>• Psychiatrist-confirmed pathological gambling / gambling disorder	• No formal diagnosis of pathological gambling / gambling disorder<br>• Current intellectual disability / intellectual developmental disorder

↓

Diagnostic assessment

Initial clinical diagnosis established by psychiatrist

SCID-5-CV current-disorders screening administered at study inclusion

Gateway item I16 used to screen for gambling involvement in the previous 12 months

Affirmative response prompted focused DSM-5-TR assessment of the 9 Gambling Disorder criteria

↓

Final study sample

Included participants: N = 122 adults

↓

Measures and analytic samples

**A. DSM-5-TR Gambling Disorder severity score**	**B. CERQ**	**C. QOLI**	**D. PCF**
9 binary criteria (0 = absent, 1 = present)<br>Total score range: 0–9<br>Severity categories: mild 4–5, moderate 6–7, severe 8–9<br>**Analytic sample: N = 122**	Cognitive Emotion Regulation Questionnaire<br>**Analytic sample: N = 122**	Quality of Life Inventory<br>**Analytic sample: N = 122**	Personality Clinical Form<br>Administered to N = 122<br>13 invalid profiles excluded from PCF-based analyses<br>**Valid PCF analytic sample: n = 109**

↓

Statistical analysis

- Complete-case, instrument-specific analyses
- Nonparametric methods due to ceiling effect and non-normal severity distribution
- Spearman's rho for correlations
- Mann–Whitney U and Kruskal–Wallis H for group comparisons
- Benjamini–Hochberg false discovery rate correction for correlation families
- Dunn–Bonferroni post-hoc tests for significant Kruskal–Wallis results
- 95% confidence intervals for Spearman's rho

Main objective: *to examine associations of DSM-5-TR gambling symptom burden with personality dimensions, cognitive emotion regulation, quality of life, and sociodemographic variables.*
