# Supplementary material for: Exploratory Associations of Personality Traits, Cognitive Emotion Regulation, and Quality of Life with DSM-Related Symptom Burden in Gambling Disorder
Source: Clin Pract. 2026 Jun 29;16(7):122. doi: 10.3390/clinpract16070122 (PMC13407655; doi:10.3390/clinpract16070122)
Supplement: Supplementary file 1 [file clinpract-16-00122-s001.zip › Supplementary Materials S2.pdf]

Supplementary Materials S2

Table S1. Item-Level Statistics and Internal Consistency of the Binary Nine-Item DSM-5-TR Severity Score (N = 122).

Note. Prevalence = proportion of participants meeting each criterion (any endorsement from Rarely to Very Often). r_it = corrected item-total correlation (Spearman). α if deleted = Cronbach's alpha when item is excluded. C1 r_it = n/a due to near-zero variance (90.2% endorsement). C6 item-total correlation non-significant (p = .096), but item retained as it constitutes a DSM-5-TR diagnostic criterion. Full scale: Cronbach's α = 0.650.

DSM-5-TR Criterion Item	Prevalence	r_it	α if deleted	p (r_it)
C1 – Need to gamble larger amounts	90.2%	.n/a	.660	< .001
C2 – Restlessness/irritability when stopping	88.5%	.318	.660	< .001
C3 – Repeated unsuccessful attempts to stop	86.9%	.344	.572	< .001
C4 – Persistent preoccupation with gambling	91.8%	.210	.628	< .001
C5 – Gambling to escape dysphoric mood	88.5%	.370	.584	< .001
C6 – Chasing losses	91.8%	.151	.661	n.s.
C7 – Lying about gambling involvement	86.1%	.298	.602	< .001
C8 – Jeopardised relationships/opportunities	88.5%	.399	.553	< .001
C9 – Reliance on others for money	84.4%	.444	.662	< .001
**Full scale (9 items, range 0–9)**	—	—	.650	—

Table S2. Spearman Correlations Between DSM-5-TR Severity Score (0–9) and PCF Scales (N = 109; FDR family: 50 tests).

Note. ρ = Spearman rank-order correlation. 95% CI via Fisher z-transformation. p = exact raw p-value. q = Benjamini–Hochberg adjusted q-value. Sig. = q ≤ .05. Green shading = significant associations. Three traits non-significant: GR (Grandiosity), RI (Risk Taking), PF (Rigid Perfectionism).

Scale / Subscale	ρ	95% CI	p	q	Sig.
**Psychopathological Tendencies (PCF)**					
IX – Emotional Problems	0.349	[0.172, 0.504]	0.0002	0.0008	**Yes***
EX – Behavioral Problems	0.388	[0.216, 0.537]	< .0001	0.0002	**Yes***
TX – Cognitive Problems	0.338	[0.161, 0.495]	0.0003	0.0011	**Yes***
**Maladaptive Personality Dimensions (PID-5)**					
NG – Negative Emotionality	0.369	[0.195, 0.521]	0.0001	0.0004	**Yes***
DM – Detachment	0.297	[0.115, 0.459]	0.0017	0.0029	**Yes***
AG – Antagonism	0.323	[0.144, 0.482]	0.0006	0.0015	**Yes***
DH – Disinhibition	0.227	[0.041, 0.398]	0.0176	0.0200	**Yes***
PT – Psychoticism	0.305	[0.124, 0.466]	0.0013	0.0025	**Yes***
**Maladaptive Traits -Significant after FDR**					

Scale / Subscale	ρ	95% CI	p	q	Sig.
AX – Anxiety	0.254	[0.069, 0.422]	0.0077	0.0098	**Yes***
DE – Depression	0.417	[0.248, 0.561]	< .0001	0.0001	**Yes***
PN – Perseveration	0.297	[0.116, 0.46]	0.0017	0.0029	**Yes***
AS – Separation Anxiety	0.243	[0.058, 0.413]	0.0108	0.0132	**Yes***
SB – Submissiveness	0.198	[0.011, 0.373]	0.0387	0.0421	**Yes***
TM – Shyness	0.266	[0.082, 0.432]	0.0052	0.0075	**Yes***
LE – Emotional Lability	0.383	[0.21, 0.533]	< .0001	0.0002	**Yes***
OT – Hostility	0.35	[0.173, 0.505]	0.0002	0.0008	**Yes***
AH – Anhedonia	0.325	[0.146, 0.483]	0.0006	0.0014	**Yes***
RS – Restricted Affectivity	0.232	[0.046, 0.403]	0.0151	0.0179	**Yes***
AP – Anhedonia (Pleasure)	0.255	[0.071, 0.423]	0.0074	0.0097	**Yes***
EA – Emotional Avoidance	0.288	[0.105, 0.451]	0.0024	0.0038	**Yes***
SP – Suspiciousness	0.252	[0.067, 0.42]	0.0082	0.0103	**Yes***
MN – Manipulativeness	0.295	[0.113, 0.457]	0.0019	0.0030	**Yes***
DU – Deceitfulness	0.354	[0.177, 0.508]	0.0002	0.0007	**Yes***
IS – Callousness	0.336	[0.157, 0.493]	0.0004	0.0011	**Yes***
CA – Attention Seeking	0.193	[0.005, 0.368]	0.0442	0.0470	**Yes***
IM – Impulsivity	0.271	[0.088, 0.437]	0.0043	0.0063	**Yes***
LR – Irresponsibility	0.301	[0.12, 0.463]	0.0015	0.0026	**Yes***
DB – Distractibility	0.329	[0.15, 0.487]	0.0005	0.0014	**Yes***
EC – Eccentricity	0.303	[0.122, 0.465]	0.0014	0.0026	**Yes***
ES – Unusual Beliefs	0.207	[0.02, 0.38]	0.0306	0.0340	**Yes***
DD – Perceptual Dysregulation	0.229	[0.043, 0.4]	0.0165	0.0192	**Yes***
GR – Grandiosity (n.s.)	0.051	[-0.138, 0.237]	0.5967	0.5967	No
RI – Risk Taking (n.s.)	0.167	[-0.021, 0.345]	0.0820	0.0855	No
PF – Rigid Perfectionism (n.s.)	0.152	[-0.037, 0.331]	0.1139	0.1162	No
**Personality Disorders (LPFS-BF)**					
TPBO – Borderline	0.408	[0.238, 0.553]	< .0001	0.0001	**Yes***
TPDP – Dependent	0.301	[0.12, 0.463]	0.0015	0.0026	**Yes***
TPOC – Obsessive-Compulsive	0.327	[0.148, 0.485]	0.0005	0.0014	**Yes***
TPPA – Paranoid	0.319	[0.139, 0.478]	0.0007	0.0016	**Yes***
TPST – Schizotypal	0.34	[0.162, 0.496]	0.0003	0.0011	**Yes***
TPSZ – Schizoid	0.258	[0.073, 0.425]	0.0068	0.0091	**Yes***

Scale / Subscale	ρ	95% CI	p	q	Sig.
TPHI – Histrionic	0.285	[0.103, 0.449]	0.0026	0.0040	**Yes***
TPNA – Narcissistic	0.265	[0.08, 0.431]	0.0054	0.0076	**Yes***
TPEV – Avoidant	0.309	[0.129, 0.47]	0.0011	0.0022	**Yes***
TPAS – Antisocial	0.377	[0.203, 0.528]	0.0001	0.0003	**Yes***
**Personality Functioning (LPFS-BF)**					
ID – Diffuse Identity	0.43	[0.263, 0.572]	< .0001	0.0001	**Yes***
SC – Impaired Goals	0.406	[0.236, 0.552]	< .0001	0.0001	**Yes***
EM – Deficient Empathy	0.333	[0.155, 0.491]	0.0004	0.0012	**Yes***
IT – Impaired Intimacy	0.318	[0.138, 0.478]	0.0007	0.0016	**Yes***
SELF – Problematic Self	0.425	[0.258, 0.568]	< .0001	0.0001	**Yes***
REL – Impaired Relationships	0.386	[0.214, 0.535]	< .0001	0.0002	**Yes***

Table S3. Spearman Correlations Between DSM-5-TR Severity Score (0–9) and CERQ Subscales (N = 122; FDR family: 9 tests).

Note. Green shading = significant after FDR correction (q ≤ .05). Three adaptive strategies (Positive Reappraisal, Putting into Perspective, Positive Refocusing) showed significant negative associations with severity.

Scale / Subscale	ρ	95% CI	p	q	Sig.
**Maladaptive Strategies**					
Catastrophizing	0.289	[0.117, 0.444]	0.0013	0.0066	**Yes***
Blaming Others	0.284	[0.112, 0.44]	0.0015	0.0066	**Yes***
Rumination	0.127	[-0.052, 0.298]	0.1647	0.2471	No
Self-Blame	0.084	[-0.095, 0.258]	0.3577	0.4024	No
**Adaptive Strategies**					
Positive Reappraisal	-0.268	[-0.426, -0.095]	0.0028	0.0066	**Yes***
Putting into Perspective	-0.267	[-0.425, -0.094]	0.0029	0.0066	**Yes***
Refocus on Planning	-0.014	[-0.191, 0.164]	0.8806	0.8806	No
Positive Refocusing	-0.205	[-0.369, -0.028]	0.0235	0.0423	**Yes***
Acceptance	0.11	[-0.069, 0.282]	0.2275	0.2925	No

Table S4. Spearman Correlations Between QOLI T-score and PCF Scales (N = 109; FDR family: 50 tests).

Note. Green shading = significant after FDR (q ≤ .05). Non-significant scales included for completeness.

Scale / Subscale	ρ	95% CI	p	q	Sig.
**Psychopathological Tendencies**					
IX – Emotional Problems	-0.325	[-0.483, -0.146]	0.0006	0.0048	**Yes***
TX – Cognitive Problems	-0.235	[-0.405, -0.049]	0.0138	0.0491	**Yes***
EX – Behavioral Problems (n.s.)	-0.056	[-0.242, 0.133]	0.5604	0.6595	No
**Maladaptive Dimensions -Significant**					
NG – Negative Emotionality	-0.284	[-0.448, -0.101]	0.0028	0.0156	**Yes***
DM, AG, DH, PT -n.s.	n.s.	[—, —]	0.1000	0.2000	No
**Maladaptive Traits -Significant after FDR**					
SB – Submissiveness	-0.366	[-0.518, -0.191]	0.0001	0.0042	**Yes***
EC – Eccentricity	-0.343	[-0.499, -0.166]	0.0003	0.0042	**Yes***
DE – Depression	-0.287	[-0.45, -0.104]	0.0025	0.0156	**Yes***
NG – Negative Emotionality	-0.284	[-0.448, -0.101]	0.0028	0.0156	**Yes***
TM – Shyness	-0.264	[-0.431, -0.08]	0.0055	0.0249	**Yes***
PN – Perseveration	-0.248	[-0.417, -0.063]	0.0093	0.0385	**Yes***
AH – Anhedonia	-0.243	[-0.413, -0.058]	0.0108	0.0415	**Yes***
TX – Cognitive Problems	-0.235	[-0.405, -0.049]	0.0138	0.0491	**Yes***
**Personality Disorders -Significant**					
TPBO – Borderline	-0.337	[-0.494, -0.159]	0.0003	0.0042	**Yes***
TPDP – Dependent	-0.273	[-0.439, -0.09]	0.0040	0.0201	**Yes***
All others -n.s.	n.s.	[—, —]	0.1000	0.2000	No
**Personality Functioning -Significant**					
ID – Diffuse Identity	-0.346	[-0.502, -0.169]	0.0002	0.0042	**Yes***
SELF – Problematic Self	-0.33	[-0.488, -0.151]	0.0005	0.0046	**Yes***

Scale / Subscale	ρ	95% CI	p	q	Sig.
SC – Impaired Goals	-0.296	[-0.458, -0.114]	0.0018	0.0128	**Yes***
EM, IT, REL -n.s.	n.s.	[—, —]	0.2000	0.4000	No

Table S5. Spearman Correlations Between QOLI T-score and CERQ Subscales (N = 122; FDR family: 9 tests).

Note. Green shading = significant after FDR (q ≤ .05).

Scale / Subscale	ρ	95% CI	p	q	Sig.
**Adaptive Strategies**					
Refocus on Planning	0.285	[0.113, 0.44]	0.0015	0.0076	**Yes***
Positive Reappraisal	0.205	[0.028, 0.369]	0.0238	0.0714	No
Acceptance	0.19	[0.013, 0.356]	0.0360	0.0809	No
Positive Refocusing	0.061	[-0.118, 0.237]	0.5012	0.5042	No
Putting into Perspective	-0.061	[-0.236, 0.118]	0.5042	0.5042	No
**Maladaptive Strategies**					
Catastrophizing	-0.281	[-0.437, -0.109]	0.0017	0.0076	**Yes***
Self-Blame	-0.174	[-0.342, 0.003]	0.0547	0.0985	No
Blaming Others	-0.12	[-0.292, 0.059]	0.1863	0.2794	No
Rumination	-0.088	[-0.262, 0.091]	0.3326	0.4276	No

Table S6. DSM-5-TR Severity Score by Sociodemographic Variables: Medians, IQRs, and Nonparametric Tests (N = 122).

Note. † Significant overall test (p < .05). Mdn = median; IQR = interquartile range. Widowed group (N = 3) and High social support group (N = 10): interpret with caution. Most groups show Mdn = 9 due to ceiling effect; group differences are captured by IQR and rank distributions.

Variable / Group	N	Mdn	IQR	Test	Statistic	p
**Sex (Mann–Whitney U)**						
Female	15	9.0	9.0–9.0		U = 838.5	p = .703
Male	107	9.0	9.0–9.0			
**Age Category (Kruskal–Wallis H)**						
25–34 years	42	9.0	9.0–9.0	H(3)	= 1.326	p = .723
35–44 years	37	9.0	9.0–9.0			
45–54 years	19	9.0	8.5–9.0			

Variable / Group	N	Mdn	IQR	Test	Statistic	p
≥ 55 years	9	9.0	7.0–9.0			
**Marital Status (Kruskal–Wallis H) -significant †**						
Single	47	9.0	9.0–9.0	H(3)	= 8.756	p = .033
Married/partnered	42	9.0	8.0–9.0			
Divorced/separated	30	9.0	9.0–9.0			
Widowed	3	9.0	8.5–9.0			
**Educational Level (Kruskal–Wallis H)**						
University	43	9.0	8.5–9.0	H(3)	= 1.814	p = .612
Post-secondary	27	9.0	9.0–9.0			
Secondary	47	9.0	9.0–9.0			
Primary	5	9.0	9.0–9.0			
**Occupational Status (Kruskal–Wallis H) -significant †**						
Full-time employed	62	9.0	8.0–9.0	H(5)	= 13.433	p = .020
Part-time employed	16	9.0	9.0–9.0			
Student	15	9.0	9.0–9.0			
Unemployed	9	9.0	9.0–9.0			
Retired	7	9.0	7.0–9.0			
Other	13	9.0	9.0–9.0			
**Income (Kruskal–Wallis H)**						
< 2,000 RON	27	9.0	9.0–9.0	H(4)	= 7.731	p = .102
2,000–3,999 RON	36	9.0	9.0–9.0			
4,000–5,999 RON	35	9.0	8.0–9.0			
6,000–9,999 RON	16	9.0	8.8–9.0			
≥ 10,000 RON	8	9.0	8.0–9.0			
**Residence (Mann–Whitney U)**						
Urban	81	9.0	9.0–9.0		U = 1782.0	p = .366
Rural	41	9.0	8.0–9.0			
**Family History (Kruskal–Wallis H)**						

Variable / Group	N	Mdn	IQR	Test	Statistic	p
First-degree relative	36	9.0	8.8–9.0	H(2)	= 5.358	p = .069
Second-degree relative	40	9.0	9.0–9.0			
No family history	46	9.0	8.0–9.0			
**Perceived Social Support (Kruskal–Wallis H) -significant †**						
Low	44	9.0	9.0–9.0	H(2)	= 11.157	p = .004
Moderate	68	9.0	9.0–9.0			
High	10	8.0	7.2–9.0			

Table S7. Dunn Post-hoc Pairwise Comparisons -Marital Status (Bonferroni-corrected, 6 pairs).

Group 1	Group 2	MR 1	MR 2	z	p (raw)	p (Bonf.)	Sig.
Single	Married	64.7	50.1	2.930	0.0034	0.0203	**Yes***
Single	Divorced	64.7	63.0	1.050	0.2936	1.0000	No
Single	Widowed	64.7	55.5	.809	0.4188	1.0000	No
Married	Divorced	50.1	63.0	-1.576	0.1150	0.6899	No
Married	Widowed	50.1	55.5	-.235	0.8138	1.0000	No
Divorced	Widowed	63.0	55.5	.390	0.6967	1.0000	No

Note. MR = mean rank. Sig. = Bonferroni-corrected p ≤ .05. Green = significant pair.

Table S8. Dunn Post-hoc Pairwise Comparisons -Social Support (Bonferroni-corrected, 3 pairs).

Group 1	Group 2	MR 1	MR 2	z	p (raw)	p (Bonf.)	Sig.
Low	Moderate	65.9	60.5	1.060	0.2894	0.8681	No
Low	High	65.9	33.8	3.340	0.0008	0.0025	**Yes***
Moderate	High	60.5	33.8	2.849	0.0044	0.0132	**Yes***

Note. MR = mean rank. High support group N = 10; interpret with caution.

Table S9. Dunn Post-hoc Pairwise Comparisons -Occupational Status (Bonferroni-corrected, 15 pairs).

Group 1	Group 2	MR 1	MR 2	z	p (raw)	p (Bonf.)	Sig.
Full-time	Part-time	54.2	73.9	-2.116	0.0343	0.5150	No
Full-time	Other	54.2	79.2	-2.485	0.0130	0.1944	No

Group 1	Group 2	MR 1	MR 2	z	p (raw)	p (Bonf.)	Sig.
Full-time	Student	54.2	70.4	-1.631	0.1029	1.0000	No
Full-time	Unemployed	54.2	68.6	-1.450	0.1469	1.0000	No
Full-time	Retired	54.2	44.1	1.014	0.3108	1.0000	No
Other pairs	—	—	—	—	1.0000	1.0000	No

Note. Overall $H(5) = 13.433$, $p = .020$. No pairwise comparison survived Bonferroni correction (15 pairs). Results reported for transparency.

Table S10. Synthesis of Results in Relation to the Study Objectives and Hypotheses

Objective	Hypothesis	Result	Conclusion
**PCF – DSM severity correlations**	Positive correlation between psychopathological tendencies, maladaptive dimensions and traits, and symptom severity	$\rho = .19–.43$, $p < .05$ for IX, EX, TX; all five dimensions (NG, DM, AG, DH, PT); 23/26 traits	Supported
	Correlation between personality disorders and symptom severity	$\rho = .26–.41$, $p < .05$ for all 10 disorders (strongest: TPBO)	Supported
	Positive correlation between personality dysfunction and symptom severity	$\rho = .32–.43$, $p < .05$ for all 6 indicators (ID, SC, EM, IT, SELF, REL)	Supported
**CERQ – DSM severity correlations**	Positive correlation between maladaptive strategies (self-blame, rumination, catastrophizing, blaming others) and symptom severity	$\rho = .28–.29$, $p < .05$ for catastrophizing and blaming others; rumination and self-blame n.s.	Partially supported
	Negative correlation between adaptive strategies (acceptance, positive refocusing, refocus on planning, positive reappraisal, putting into perspective) and symptom severity	$\rho = −.21$ to $−.27$, $p < .05$ for positive reappraisal, putting into perspective, positive refocusing; acceptance and refocus on planning n.s.	Partially supported
**PCF/CERQ – QOLI correlations**	Negative correlation between PCF scores and quality of life	$\rho = −.24$ to $−.37$, $p < .05$ for IX, TX, NG, six traits, TPBO, TPDP, ID, SC, SELF; other scales n.s.	Partially supported
	Negative correlation between maladaptive CERQ strategies and quality of life	$\rho = −.28$, $p < .05$ only for catastrophizing; others n.s.	Partially supported
	Positive correlation between adaptive CERQ strategies and quality of life	$\rho = .29$, $p < .05$ only for refocus on planning; others n.s.	Partially supported
**Demographic differences – DSM severity**	Significant differences based on sociodemographic variables	$p < .05$ for marital status ($H(3) = 8.76$, $p = .033$) and occupational status ($H(5) = 13.43$, $p = .020$; omnibus only, no pair surviving Bonferroni); sex, age, education, income, residence n.s.	Partially supported
	Significant differences based on family history	$H(2) = 5.358$, $p = .069$	Not supported
	Significant differences based on perceived social support	$H(2) = 11.157$, $p = .004$ (Low > High and Moderate > High after Bonferroni)	Supported
