# Supplementary material for: Exploratory Associations of Personality Traits, Cognitive Emotion Regulation, and Quality of Life with DSM-Related Symptom Burden in Gambling Disorder
Source: Clin Pract. 2026 Jun 29;16(7):122. doi: 10.3390/clinpract16070122 (PMC13407655; doi:10.3390/clinpract16070122)
Supplement: Supplementary file 1 [file clinpract-16-00122-s001.zip › Supplementary Materials S3.pdf]

Supplementary Materials S3 - Personality Clinical Form (PCF) scales and abbreviations

PCF scale group	Abbreviation	Scale / indicator label
**Psychopathological tendencies (PCF derived scales)**	IX	Emotional Problems
	EX	Behavioral Problems
	TX	Cognitive Problems
**Maladaptive personality domains (PID-5-related PCF domains)**	NG	Negative Emotionality
	DM	Social and Affective Detachment
	AG	Antagonism
	DH	Disinhibition
	PT	Psychoticism
**Maladaptive trait scales (PCF primary clinical scales)**	AX	Anxiety
	DE	Depression / Depressivity
	PN	Negative Perseveration / Perseveration
	AS	Separation Anxiety
	SB	Submissiveness
	TM	Shyness
	LE	Emotional Lability
	OT	Hostility
	AH	Anhedonia
	RS	Social Withdrawal
	AP	Affective Flattening / Restricted Affectivity
	EA	Avoidance of Intimacy
	SP	Suspiciousness
	MN	Manipulativeness
	DU	Deceitfulness
	IS	Callousness / Insensitivity
	CA	Attention Seeking
	IM	Impulsivity
	LR	Irresponsibility
	DB	Distractibility
	EC	Eccentricity
	ES	Unusual Experiences and Bizarre Beliefs
	DD	Cognitive and Perceptual Dysregulation
	GR	Grandiosity
	RI	Risk Taking
	PF	Rigid Perfectionism
**Personality-disorder feature scales (PCF categorical feature scales)**	TPBO	Borderline
	TPDP	Dependent
	TPOC	Obsessive-Compulsive
	TPPA	Paranoid
	TPST	Schizotypal
	TPSZ	Schizoid
	TPHI	Histrionic
	TPNA	Narcissistic
	TPEV	Avoidant

Supplementary Table S2 – PCF scales and abbreviations

PCF scale group	Abbreviation	Scale / indicator label
	TPAS	Antisocial
**Personality functioning indicators (LPFS-BF-related PCF indicators)**	ID	Diffuse Identity
	SC	Impaired Goals
	EM	Deficient Empathy
	IT	Impaired Intimacy
	SELF	Problematic Self
	REL	Impaired Relationships

***Note.**. The PCF was used for dimensional clinical characterization of personality-related variables; personality-disorder feature scales should not be interpreted as stand-alone personality-disorder diagnoses. The personality-disorder feature scales are PCF categorical feature scales, whereas ID, SC, EM, IT, SELF, and REL are LPFS-BF-related personality-functioning indicators. GR, RI, and PF did not remain significant after Benjamini–Hochberg FDR correction; the other scales listed below remained significant after FDR correction.*

***Terminology note.** The English labels were harmonized with the manuscript terminology and the PCF nomenclature. In particular, AH denotes Anhedonia; RS denotes Social Withdrawal; AP denotes Affective Flattening / Restricted Affectivity; EA denotes Avoidance of Intimacy; and ES/DD were expanded to reflect the PCF descriptions of unusual experiences/bizarre beliefs and cognitive-perceptual dysregulation.*

***Source note.** PCF scale grouping and abbreviations follow the PCF output structure described in the manuscript and the PCF documentation cited in the main article. Statistical status is based on the manuscript results after Benjamini–Hochberg FDR correction.*
